# Purification and transcriptomic characterization of proliferative cells of *Mesocestoides corti* selectively affected by irradiation

**DOI:** 10.3389/fpara.2024.1362199

**Published:** 2024-03-05

**Authors:** Alicia Costábile, María Fernanda Domínguez, Inés Guarnaschelli, Matías Preza, Uriel Koziol, Estela Castillo, José F. Tort

**Affiliations:** ^1^Sección Bioquímica, Facultad de Ciencias, Universidad de la República, Montevideo, Uruguay; ^2^Departamento de Genética, Facultad de Medicina, Universidad de la República, Montevideo, Uruguay; ^3^Sección Biología Celular, Facultad de Ciencias, Universidad de la República, Montevideo, Uruguay; ^4^Unidad de Biología Parasitaria, Facultad de Ciencias- Instituto de Higiene, Universidad de la República, Montevideo, Uruguay

**Keywords:** cestodes, germinative cells, proliferation, irradiation, cell purification, stem cell markers, transcriptomics

## Abstract

Flatworms depend on stem cells for continued tissue growth and renewal during their life cycles, making these cells valuable drug targets. While neoblasts are extensively characterized in the free-living planarian *Schmidtea mediterranea*, and similar stem cells have been characterized in the trematode *Schistosoma mansoni*, their identification and characterization in cestodes is just emerging. Since stem cells are generally affected by irradiation, in this work we used this experimental approach to study the stem cells of the model cestode *Mesocestoides corti*. We found that gamma irradiation produces a dose-dependent decrease in proliferative cells, requiring higher doses than in other flatworms to completely abolish proliferation. The treatment results in the downregulation of candidate marker genes. Transcriptomic studies reveal that several genes downregulated after irradiation are conserved with other flatworms, and are related to cell cycle, DNA replication and repair functions. Furthermore, proliferative cells were isolated by cell sorting and also characterized transcriptomically. We found that the set of genes characteristic of proliferative cells agrees well with those downregulated during irradiation, and have a significant overlap with those expressed in planarian neoblasts or *S. mansoni* stem cells. Our study highlights that conserved mechanisms of stem cell biology may be functional in flatworms, suggesting that these could be relevant targets to evaluate in the control of parasitic species.

## 1 Introduction

Parasitic flatworms (Platyhelminthes), including cestodes and trematodes, have a huge impact on human health and production. The most relevant cestode diseases worldwide include cysticercosis caused by *Taenia solium* and hydatidosis caused by species of the genus *Echinococcus*, which have high incidence in Latin America (Garcia et al., 2007; Webb and Cabada, 2017). These neglected diseases often occur with other co-endemic diseases such as malaria, tuberculosis and AIDS, frequently resulting in multiple infections that complicate the clinical presentation and morbidity (Hotez et al., 2008). The economic burden of flatworm derived diseases is also enormous, since the main productive animal species can be infected, resulting in direct or indirect losses. A characteristic of these diseases is their chronicity, with parasites remaining in their respective hosts for many years, in some cases without clinical manifestations, and infected people and/or animals acting as reservoirs for the transmission of diseases. Although there are effective drugs for their treatment, they can cause hepato-toxicity, and generally do not prevent re-infection. Extensive use of drugs such as praziquantel and benzimidazoles have resulted in the emergence of drug resistance (Brennan et al., 2007), stressing the need for new control mechanisms, identifying new possible targets for vaccine or drug design. For this reason, it is important to deepen our knowledge of cellular and molecular aspects of parasite development and proliferation.

Flatworms depend on constant cell proliferation both for development and tissue renewal. Differentiated cells are postmitotic and a pool of undifferentiated stem cells is the source of new cells during tissue renewal, growth and regeneration (Peter et al., 2004; Reuter and Kreshchenko, 2004; Rink, 2013; Koziol and Brehm, 2015; Zhu and Pearson, 2016). These stem cells have been denominated neoblasts in many flatworm species, or germinative cells in cestodes. This cell renewal strategy differs from other animals, in which depending on the tissue, proliferating differentiated cells can coexist with tissue-specific stem cells and postmitotic differentiated cells (Peter et al., 2004). These undifferentiated cells have been extensively studied in planaria (free-living flatworms), and more recently several studies have been conducted in trematodes (Collins et al., 2013, 2016; Wang et al., 2013, 2018; Wendt et al., 2018, 2020; Diaz Soria et al., 2020; Li et al., 2021, 2018) and cestodes (Koziol et al., 2010; Cheng et al., 2017a, Cheng et al., 2017b; Domínguez et al., 2022, 2014; Schubert et al., 2014; Rozario et al., 2019). Benzimidazoles used for treatment are not efficient in eliminating these cells in the cestode *Echinococcus multilocularis*, since the beta-tubulin isoform most expressed in this cell type has less affinity for the drug (Schubert et al., 2014; Koziol and Brehm, 2015). Consequently, the development of drugs that target essential mechanisms for this cell type would eliminate the proliferative capacity of the parasite, ending the infection and preventing the parasite from recurring at the end of treatment.

Proliferative cells are affected by X and γ radiation (which produce DNA damage), leading for example to the depletion of neoblasts and impairment of tissue regeneration in planarians with doses greater than 30 Gy (Eisenhoffer et al., 2008; Wagner et al., 2012; Sahu et al., 2021). Lethal doses seem to be higher in parasitic species since reduction in the number of proliferative cells, but not ablation, delayed growth and less proliferation can be observed with doses up to 200 Gy (see Discussion, **Supplementary Table 1**). The use of sublethal doses allowed the characterization of structural and functional alterations, and the identification of downregulated genes upon irradiation in schistosomes (Collins et al., 2013, 2016; Wendt et al., 2018), and in the cestodes *E. multilocularis* (Pohle et al., 2011; Koziol et al., 2014)*, Echinococcus granulosus* (Alam-Eldin and Badawy, 2015) and *Hymenolepis diminuta* (Rozario et al., 2019) (**Supplementary Table 1**).

We and others have been analyzing different aspects of host-parasite interaction and development in the model cestode *Mesocestoides corti* (Britos et al., 2000; Markoski et al., 2003; Lalanne et al., 2004; Espinoza et al., 2005; Domínguez et al., 2007; Espinoza et al., 2007; Koziol et al., 2008, 2009, 2011; Laschuk et al., 2011; Costa et al., 2015; Costábile et al., 2017; Camargo de Lima et al., 2018; Costábile et al., 2018; Basika et al., 2019, 2020; Camargo da Lima et al., 2020; Domínguez et al., 2022). This organism is a good laboratory model since it can be easily maintained in culture and strobilar development can be induced *in vitro*, starting from larvae obtained from experimentally infected mice (Specht and Voge, 1965; Voge and Seidel, 1968; Barrett et al., 1982; Thompson et al., 1982; Ong and Smyth, 1986). We have previously shown that proliferative cells in *M. corti* are distributed through the medullary parenchyma, mostly in the peripheral region next to the inner muscular layer. In the larval stage, there is an antero-posterior gradient, being more abundant in the scolex and neck, where new segments are originated during strobilization. During segmentation, there is an accumulation of proliferative cells in the center of each proglottid forming the genital primordium (Koziol et al., 2010). These characteristics are similar to those observed for other cestodes.

Purified proliferative cells from *M. corti* show characteristic small size with large nucleus and scarce cytoplasm (Domínguez et al., 2014) similar to neoblasts from other flatworms (Baguñà and Romero, 1981; Peter et al., 2004; Rossi et al., 2008; Collins et al., 2013; Koziol et al., 2014), and can be maintained in culture for short periods (Domínguez et al., 2022).

In this work, we analyzed the effect of different doses of γ-radiation on *M. corti* proliferative cells, using cellular and molecular approaches in order to validate this technique for studying *M. corti* proliferative cells. We also performed transcriptomic analysis of irradiated and control worms, as well as from FACS-purified proliferative cells. Our results show that there is indeed a decrease in proliferation in *M. corti* after irradiation, associated with downregulation of genes associated with genetic information processes, in agreement with their upregulation in purified proliferative cells.

## 2 Materials and methods

### 2.1 Parasite culture and irradiation

*M. corti* tetrathyridia were obtained as previously described (Britos et al., 2000; Koziol et al., 2010) from infected mice kindly provided by Jenny Saldana (Laboratorio de Experimentación Animal, Facultad de Química, Universidad de la República, Uruguay). Briefly, worms were recovered aseptically with a Pasteur pipette from the peritoneal cavity and transferred to Hank’s balanced salt solution (HBS5) supplemented with 50 mg/ml gentamicin, and stored at 4°C. Worms were cultured in modified RPMI medium (10.4 g/L RPMI, 4.2 g/L NaHCO3, 4.3 g/L glucose, 4.8 g/L yeast extract and 50 µg/mL gentamicin) supplemented with Fetal Bovine Serum (10%) and sodium taurocholate (1 mg/ml), with media exchange every 48 or 72 hours.

Parasites cultured for 6 days were washed with fresh media and irradiated with doses of 100, 500 and 1000 Gy in a GammaCell 220 (MDS Nordion) at Instituto Nacional de Donación y Trasplante (Hospital de Clínicas, Universidad de la República). After irradiation, worms were allowed to recover for 1 or 5 days in culture. Control unirradiated worms were treated under identical conditions, except for the irradiation step. All experiments were performed in triplicates.

### 2.2 EdU labeling and quantification of proliferative cells

Worms were incubated with 20 µM EdU (5-Ethynyl-2´-deoxyuridine, Invitrogen) in modified RPMI media without yeast extract for 4 hs, washed twice with PBS, incubated in ice for 10 min and fixed in 4% paraformaldehyde in PBS preheated to 90°C. Worms were fixed overnight at 4°C, dehydrated by incubation in 100% methanol for 10 minutes and stored in 100% methanol at -20°C. For detection, samples were re-hydrated progressively and incubated for 30 min in 3% BSA in PBS at room temperature. Washing and detection were performed with Click-iT EdU Imaging kit (Invitrogen) according to manufacturer instructions. Nuclei were stained with 2 μg/mL DAPI in PBS for 15 min at room temperature after EdU detection. Images were taken with confocal microscopy (Zeiss LSM 800, Advanced Bioimaging Unit, Institut Pasteur de Montevideo). For the quantification of EdU^+^ cells, single plane confocal sections of five random parasites per condition were selected. Counts were performed using Fiji (Schindelin et al., 2012), including thresholding of EdU signal and counting positive nuclei with the “Analyze Particles” tool. Counts were normalized by area (EdU^+^ cells/mm^2^) and averaged. Mann-Whitney test was used for statistical analysis.

### 2.3 Real time PCR of marker genes

RNA was extracted using Trizol (Ambion), according to manufacturer instructions. DNase treatment (TURBO DNase, Ambion), quantification (Qubit RNA Broad Range, Invitrogen) and reverse transcription using 500 ng total RNA and random primers (SuperScript II, Invitrogen), were done as previously published (Costábile et al., 2017).

mRNA levels of candidate proliferative cells marker genes and the endogenous reference gene GAPDH were analyzed by RT-qPCR. Primers were designed using Primer3plus (Untergasser et al., 2007) (**Supplementary Table 2**). qPCR reactions were performed with QuantiNova SYBR Green PCR kit (Qiagen) in a Step One Plus Real Time PCR system (Applied Biosystems) with fast-cycling conditions (95°C for 2 min followed by 40 cycles of 95°C for 5 sec and 60°C for 10 sec). Melting curve analysis was performed after amplification with the equipment default program. Primer efficiencies were determined using serial dilutions of a pool of all cDNAs used in the analysis according to Costábile et al. (2017). mRNA fold change for each gene was determined using the 2^-ΔΔCt^ method (Livak and Schmittgen, 2001) with non-irradiated worms as reference condition and GAPDH as endogenous control gene. Statistical analysis on ΔΔCt values were performed with ggpubr (v0.2, Kassambara, 2018) R package (Kruskal-Wallis test). The p-values were adjusted using Holm correction.

### 2.4 RNA sequencing of irradiated and unirradiated worms

RNA extracted from unirradiated and irradiated worms (100 Gy after 1 day of recovery) using *miRvana* (*Ambion*) was treated with TURBO DNase (Ambion) and quantified using Qubit RNA Broad Range kit (Invitrogen), and sent in dry ice to Beijing Genomics Institute (Shenzhen, China) for sequencing. Libraries were prepared with TruSeq™ RNA Sample Preparation kit (Illumina), starting with polyA-purified RNA and 100 PE sequencing in an Illumina HiSeq 2000. The data have been deposited with links to BioProject accession number PRJNA950029 in the NCBI BioProject database (https://www.ncbi.nlm.nih.gov/bioproject/).

### 2.5 Fluorescence activated cell sorting and RNA extraction

Purified proliferative cells were obtained from single cell suspensions of 6-days cultured tetrathyridia of *M.corti* according to Domínguez et al. (2014). Briefly, chopped worms were treated with 0.1% trypsin in modified RPMI with 2mM EDTA, using mild agitation in a magnetic stirrer and pipetting to obtain isolated cells. Live cells were filtered through 45μm pore-size gauze and stained for flow cytometry (0.02 μg/ml of Calcein AM in modified RPMI with 2mM EDTA for 90 min at 20°C and 5 μg/ml of Hoechst 33342 in PBS with 2mM EDTA for 20 min at 37° C and 2μg/ml Propidium Iodide immediately before sorting). Flow cytometric analysis and cell sorting was performed using a High-Speed cell sorter MoFlo (Beckman Coulter)with a 70-nm nozzle at a rate of 500–1500/eps. Sorting mode was set in Sort purify 1–2 drops and sorting decision was based on: FL 1/FL 2 plots to determine viable cells (Calcein AM positive/propidium iodide negative cells), FL 7/FL 8 Hoechst 33342 pulse analysis for doublet discrimination and G2/M selection and G1/G0 selection [for further details, see Domínguez et al. (2014)]. Cells in S/G2/M (~10^5^ cells per run) and G0/G1 (~10^6^ cells per run) phases of cell cycle were collected in 10mL PBS with 3% BSA and centrifuged for 10 minutes at 4000 rpm. The supernatant was re-centrifuged in the same conditions. Cell pellets were resuspended in 100 µL Trizol (Ambion) and stored at -80°C.Three runs of FACS were pooled, and RNA was extracted with Direct-zolTM RNA MiniPrep kit (Zymo Research), obtaining on average 125 ng of total RNA from cells in S/G2/M phases and 1µg from cells in G0/G1 phases.

### 2.6 RNA sequencing of isolated cells

Libraries were constructed locally using NEBNext Ultra Directional library kit (New England Biolabs, Catalog N°: E7420) with the poly-A purification module. Sixty ng of total RNA were used for each indexed library as a starting point. Libraries were paired-end sequenced (150 nt) in a HiSeq2500 equipment (Illumina) at Novogene (Beijing, China). The data have been deposited with links to BioProject accession number PRJNA1039817 in the NCBI BioProject database (https://www.ncbi.nlm.nih.gov/bioproject/).

### 2.7 Read quality, mapping and differential expression

Reads were trimmed to eliminate adapters and low quality bases using Trimmomatic (v 0.33, Bolger et al., 2014), with the parameters HEADCROP:8 ILLUMINACLIP : TruSeq2-PE.fa:2:30:10 LEADING:3 TRAILING:3 SLIDINGWINDOW:4:15 MINLEN:36. Trimmed paired and unpaired reads were mapped to *M. corti* genome assembly (downloaded from WormBase Parasite: https://ftp.ebi.ac.uk/pub/databases/wormbase/parasite/releases/WBPS1/species/mesocestoides_corti/PRJEB510/mesocestoides_corti.PRJEB510.WBPS1.genomic.fa.gz) using hisat (v2.1.0, Kim et al., 2015) with default parameters. Gene models were downloaded from WormBase ParaSite (https://ftp.ebi.ac.uk/pub/databases/wormbase/parasite/releases/WBPS14/species/mesocestoides_corti/PRJEB510/mesocestoides_corti.PRJEB510.WBPS14.genomic.fa.gz) and used to calculate read counts for each gene using the featureCounts tool of the R package Rsubread (v2.4.3, Liao et al., 2019). Differential expression analysis was performed using DESeq2 package (v1.30.1, Love et al., 2014) using count data. Genes with |Fold Change| >1.5 and adjusted p-value <0.05 were considered differentially expressed. Heatmap of differentially expressed genes was done with the R package pheatmap (v1.0.12, Kolde, 2019), using pseudocounts (log2(counts+1). Protein annotation was obtained from Uniprot (https://www.uniprot.org/uniprotkb?query=(proteome:UP000046399, downloaded 06/23/2023).

Regularized log transformation of normalized counts for each gene in each condition were used for PCA analysis and plotted with ggplot2 package (v3.1.0, Wickham, 2016).

### 2.8 Metabolic pathway and gene ontology enrichment analysis

For enrichment analysis, one representative isoform per gene was selected, based on annotation and protein and transcript length. Protein sequences of differentially expressed genes in proliferative and differentiated cells were analyzed for pathway enrichment using KOBAS3.0 with default parameters (Xie et al., 2011) (online version: http://kobas.cbi.pku.edu.cn/genelist/). *Homo sapiens* was selected as the reference organism for analysis of KEGG pathways and the Reactome database. GO terms for each annotated gene were retrieved from uniprot annotation. For Gene Ontology enrichment, differentially expressed genes in each condition were analyzed with R package GOSeq (v2.34.0, Alexa and Rahnenfuhrer, 2016). Enriched GO terms were visualized using REVIGO (Supek et al., 2011), and the classification of all annotated proteins was performed with eggNOG mapper (http://eggnog-mapper.embl.de/), with default parameters except for e-value that was set to 1e-5 (Huerta-Cepas et al., 2019).

### 2.9 Analysis of neoblast conserved genes

A list of genes associated with neoblast-like cells or downregulated upon irradiation was made based on the literature (**Supplementary Table 3**). Selected query genes correspond to those differentially expressed in *S. mansoni* neoblast-like cells, present in neoblasts clusters or differentiated cells progenitors in *S. mediterranea* or those downregulated by irradiation in *H. diminuta.* Orthologs to *M. corti* genes were identified either if (1) they were directly reported as orthologs in WormBase Parasite, or (2) they were retrieved by best reciprocal BLAST hit (e-values <e-10) with different *S. mediterranea* transcriptomes or *S.mansoni* and *H.diminuta* proteomes. Rosetta Stone Transcript Mapper (Nowotarski et al., 2021) was used for mapping gene identifiers between different *Schmidtea mediterranea* transcriptomes (**Supplementary Table 3**). A gene was considered a stem cell marker if it is reported in at least one work for *S. mansoni* or *S. mediterranea* and if it is downregulated in irradiated *H. diminuta*. Venn diagram representation of the conserved genes was done using DeepVenn (Hulsen, 2022).

## 3 Results

### 3.1 Decreased worm survival after γ-radiation is associated with a reduction in the number of proliferating cells

**Figure 1:**
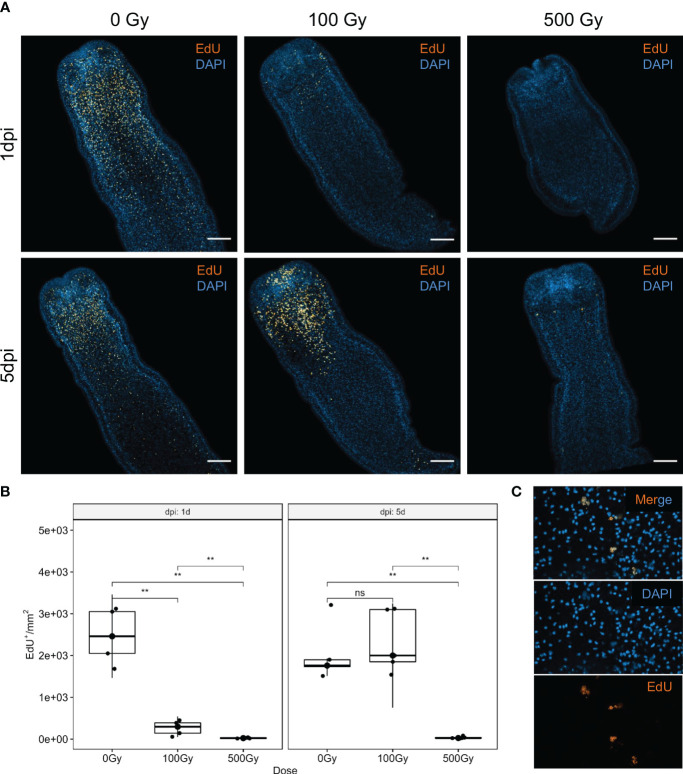
S-phase cell labeling of irradiated worms at different times after irradiation. **(A)** EdU labeling (orange) and nuclear stain (blue) of control (0 Gy) and irradiated worms with 100 Gy and 500 Gy, after one (1dpi) and five (5dpi) days after irradiation. Bar represents 100µm. **(B)** Quantification of EdU^+^ cells. **Mann-Withney p-value < 0.01. ns, non significant. Box plot indicates median and interquartile range. **(C)** Labeled nuclei are fragmented in worms irradiated with 500 Gy, after 5 days of recovery.

Irradiation of worms with different doses of γ-radiation, showed that while viability is not affected immediately, tegumental damage and swelling can be observed after 1 day in those exposed to high doses (500 Gy). However, worms irradiated with 100 Gy conserved high motility along the experiment and started to show some tegumental damage only after 4 days of *in vitro* culture (**Supplementary Figure 1**). The incorporation of the thymidine analog EdU was used to analyze the effect of radiation on proliferation. After 1 day of recovery, irradiated worms incorporate significatively less EdU than control worms (**Figure 1A**). However, worms irradiated with 100 Gy restored the amount of EdU+ cells/mm2 to a similar level than unirradiated worms after 5 days of recovery. In contrast, worms irradiated with 500 Gy almost do not incorporate EdU after 5 days, and the few labeled nuclei appear fragmented, indicating extensive DNA damage (**Figures 1B, C**). These results suggest that 100 Gy is a sub-lethal dose for *M. corti*, and in these conditions a small fraction of proliferating cells persists, and are able to proliferate and restore the proliferating cell population after 5 days of *in vitro* culture.

### 3.2 Expression of marker genes PCNA and Nanos is reduced by irradiation

**Figure 2:**
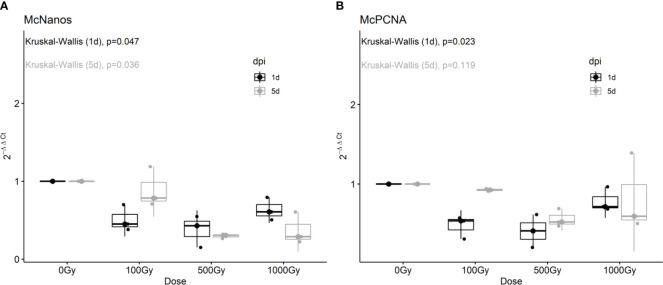
Fold change of *McNanos* **(A)** and *McPCNA* **(B)** expression in irradiated worms at different recovery times. Box plots of three replicates per condition. Results after one day post irradiation (1 dpi) are shown in black, while results after 5dpi are shown in gray. p-values from Kruskal-Wallis non-parametric test are shown above each plot.

To further characterize the effect on proliferative cells we analyzed by RT-qPCR the expression of putative neoblast marker genes in response to different doses of radiation and one and five days post-treatment. The expression of both *McNanos* and *McPCNA* diminished with all doses analyzed at the first day post-treatment, but was restored after 5 days for the 100 Gy treated worms (**Figure 2**).

These results are in agreement with EdU labeling shown previously, suggesting that at low doses proliferative cells remained viable and were able to repopulate the worm. Other candidate marker genes tested like *Pumilio* and *pL10 DEAD/box helicase* did not show significant differences in gene expression after irradiation, for neither dose applied or recovery time (**Supplementary Figure 2**), and we have recently shown that these genes are not only expressed in proliferative cells, but also in differentiated tissues (Domínguez et al., 2022).

### 3.3 Most of the downregulated genes upon irradiation are associated with DNA metabolism and the cell cycle, and conserved within flatworms

**Figure 3:**
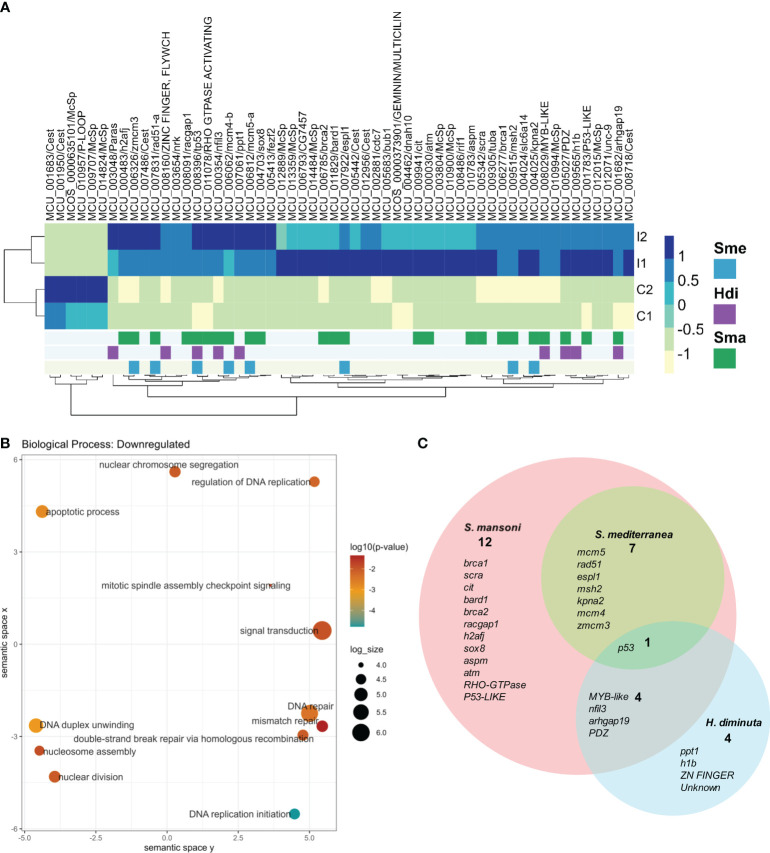
Differentially expressed genes in irradiated vs control worms. **(A)** Heatmap of normalized counts of differentially expressed genes. Gene names are shown as gene id and annotation based on BLAST results against the SwissProt database (lowercase) or InterPro database (uppercase). When no information is available, orthology results are indicated: Paras: orthologous in parasitic flatworms, Cest: orthologous in Cestodes, McSp: no orthologous in other flatworms. Genes are annotated based on conservation with orthologous genes expressed in stem cells of other flatworms: Blue: Expressed in *S. mediterranea* (Sme) neoblasts, Purple: Downregulated by irradiation in *(H) diminuta* (Hdi), Green: Expressed in *S. mansoni* (Sma) stem cells. **(B)** GO enrichment analysis of downregulated genes. Circles are sized according to log size and colored by p-value (blue - orange: lower to higher). For further details, see **Supplementary Table 5**. **(C)** Venn diagram of *M. corti* downregulated genes orthologs to genes associated with stem cells in other flatworms. Gene annotation is shown for each set. Lower case: SwissProt best hit, Upper case: InterPro conserved domain.

RNAseq of one day post treatment (100 Gy) and control worms were performed to further analyze the effects of sublethal irradiation. An average of 100 million reads per sample were obtained, with more than 95% of the good quality reads mapping to the genome (**Supplementary Table 4**). Differential expression analysis resulted in six genes upregulated and 50 genes downregulated after irradiation (**Figure 3A**).

Only one of the six upregulated genes (MCU_010957) has some annotation (with a conserved domain associated with a NTPAse), being two of the remaining unannotated genes conserved in cestodes, and other *M.corti* specific gene (MCU_009707) is one of the most expressed in the larvae. The 50 downregulated genes, expected to be expressed by proliferative cells, included 38 annotated genes, 5 unannotated genes conserved in flatworms and 7 *M. corti* specific genes. GO enrichment analysis of the annotated genes revealed terms related to DNA metabolism (recombination, replication and repair), response to X-rays, regulation of cell cycle and apoptosis, among others (**Figure 3B** and **Supplementary Table 5**). These processes are expected to be active in proliferative cells, and indeed are enriched in planarian neoblasts (Eisenhoffer et al., 2008; Galloni, 2012; Labbé et al., 2012).

Interestingly, more than half of the *M. corti* downregulated genes are orthologs of genes associated with planarian neoblasts (Fincher et al., 2018; Plass et al., 2018; Zeng et al., 2018), *S. mansoni* neoblast-like cells (Collins et al., 2013, 2016; Wang et al., 2013, 2018; Diaz Soria et al., 2020; Li et al., 2021, 2018; Wendt et al., 2018, 2020) or *H. diminuta* stem cells (Rozario et al., 2019) (**Figure 3C** and **Supplementary Table 6**). p53 is the only gene associated with stem cells in the four species, while other genes involved in processes like replication, DNA repair and cell division are conserved between diverse sets of neoblast-like cells. The remaining 22 downregulated genes are *M. corti* specific, or their orthologs genes are not associated with stem cells in the analyzed species.

### 3.4 Isolated proliferative cells have a distinct transcriptional profile

The restricted list of differentially expressed genes in irradiated worms might be related to the fact that only approximately 7% of the worm cells are proliferating (Domínguez et al., 2014). In order to further characterize proliferative cells, we decided to purify them by fluorescent activated cell sorting (FACS) based on their DNA content. Proliferative cells in S/G2/M phases of cell cycle have doubled the amount of DNA compared to cells in G0/G1 phases of the cell cycle (mostly consisting of postmitotic differentiated G0 cells, and a minor proportion of G1 proliferating cells). RNA extracted from three pooled FACS experiments was used to generate sequencing libraries of the two cell populations, obtaining between 39 and 85 million reads. Almost 80% of the reads passed the quality and adapter filters, and between 50 and 70% mapped to the genome. On average we obtained 38 and 32 million good quality reads for G0/G1 samples and G2/S/M samples respectively (**Supplementary Table 7**).

**Figure 4:**
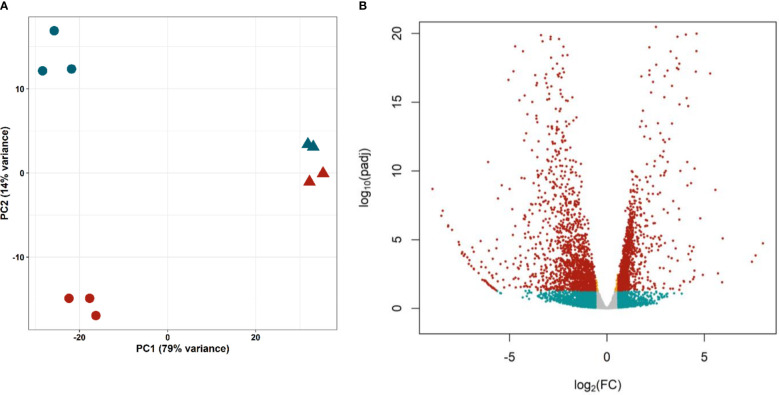
Differential expression in isolated proliferative and differentiated *M. corti* cells. **(A)** Principal Component Analysis of purified cells in different phases of cell cycle (S/G2/M, blue circles, G0/G1 red circles) and irradiated (red triangles) and control (blue triangles) worms. **(B)** Volcano plot comparing proliferating (S/G2/M) cells vs differentiated (G0/G1) cells. Orange dots: significant genes with |FC| <1.5. Blue dots: not significant genes with |FC| >1.5. Red dots: Significant genes with |FC| >1.5.

Principal Component Analysis (**Figure 4A**) showed a good separation of cell samples in different proliferation states (G0/G1 and S/G2/M, circles), distant on the first component to whole worm samples (triangles). The short distance between control and irradiated worms might be explained by the low proportion of proliferating cells. In any case, the second component separates samples containing proliferating cells (control worms and G2/S/M samples, blue) from samples without them (irradiated worms and G0/G1 samples, red), confirming that the technique optimized by Domínguez et al. (2014) can purify cells with different gene expression profiles.

Differential expression analysis resulted in 2688 genes with significant differences in expression levels between proliferating and differentiated cells (adjusted p-value <0.05, **Figure 4B** and **Supplementary Table 8**). Of these, 1230 genes are upregulated (Fold Change >1.5) in proliferative cells (G2/S/M population), while 1485 are downregulated (Fold Change < -1.5), and are mostly expressed in differentiated cells (G0/G1 population).

### 3.5 DNA metabolism, replication and gene expression are pathways enriched in proliferative cells

**Figure 5:**
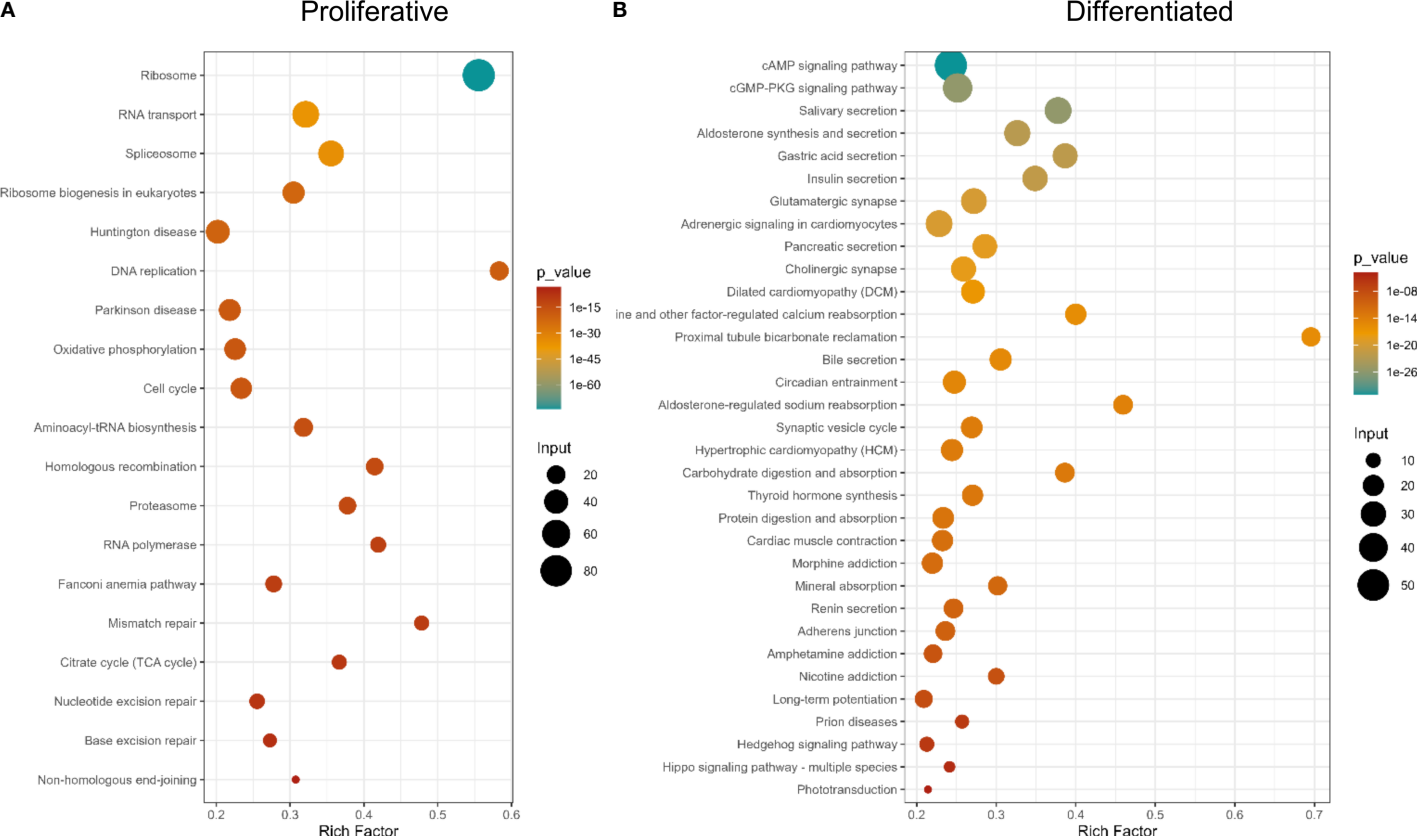
KEGG Pathway enrichment analysis of differentially expressed genes. **(A)** Genes up-regulated in proliferative cells, **(B)** Genes up-regulated in differentiated cells.

KEGG pathway analysis showed gene expression and DNA metabolism pathways enriched in proliferative cells, while pathways associated with digestion, excretion, nervous system and signal transduction, among other terms, being associated with differentiated tissues (**Figure 5** and **Supplementary Table 9**). GO enrichment analysis showed more terms enriched in proliferative cells [21 in Biological Process (BP), 21 Cellular Compartment (CC), and 22 Molecular Function (MF)] than in differentiated cells (BP: 1, CC: 5, MF: 3, [**Supplementary Figure 3**, and **Supplementary Table 9**)]. This is expected, since differentiated cells have several different functions depending on cell specialization. Since proliferative cells were selected based on their replication ability, enriched GO terms associated with DNA replication and repair, translation, RNA metabolism and cell cycle and similar KEGG enrichment pathways are expected.

When analyzing protein functional categories with EggNOG database, in differentially expressed genes, the same functions are more expressed in proliferative cells (**Supplementary Figure 3** and **Supplementary Table 9**). Also, the functional category “Energy production and conversion” is more enriched in proliferative cells, compared to differentiated cells. Consistent results were obtained using the Reactome database (**Supplementary Table 9**).

### 3.6 Most of the genes downregulated in irradiated worms are differentially expressed in purified proliferative cells

Forty-one of the 50 genes downregulated in irradiated worms are also differentially expressed in proliferative cells (**Table 1**), consistent with the idea that irradiation affects mainly proliferative cells. Almost all genes have concordant expression patterns (downregulated in irradiated worms and upregulated in proliferative cells), as only one gene, (innexin) has the opposite behavior (being downregulated in both irradiated worms and proliferative cells). Of the remaining 9 non coincident genes, three (two p53-like genes and BUB1 kinase) are highly expressed in proliferative cells, although not significantly (0.05<adjusted p-value<0.1), and one (nfil3) is most expressed in differentiated cells, but also not significantly (0.05<adjusted p-value<0.1).

**Table 1:** Differentially expressed genes in irradiated worms and upregulated in purified proliferative cells.

Gene	Irradiated		Purified cells						
	FC I/C^a^	APV^b^	FCP/D^c^	APV^b^	Annotation	Associated GO terms	Sma^1^	Hdi^2^	Sme^3^
Downregulated in irradiated worms & differentially expressed in purified cells									
MCU_012889	-3,88	0,0465	2,16	0,0246	*M. corti* Specific		0	0	0
MCU_014484	-3,73	0,0101	3,61	0,0000	*M. corti* Specific		0	0	0
MCU_006277	-2,74	0,0101	19,30	0,0119	Breast cancer type 1 susceptibility protein homolog	DNA repair [GO:0006281]	1	0	0
MCU_005342	-2,73	0,0002	24,33	0,0000	Anillin		1	0	0
MCU_009941	-2,59	0,0001	14,12	0,0000	Citron Rho-interacting kinase	ATP binding [GO:0005524]; protein kinase activity [GO:0004672]; protein phosphorylation [GO:0006468]	1	0	0
MCU_000030	-2,56	0,0098	7,55	0,0000	Serine-protein kinase ATM	membrane [GO:0016020]; protein serine/threonine kinase activity [GO:0004674]; DNA repair [GO:0006281]; phosphorylation [GO:0016310]	1	0	0
MCU_011829	-2,54	0,0036	22,74	0,0000	BRCA1-associated RING domain protein 1	metal ion binding [GO:0046872]; transferase activity [GO:0016740]	1	0	0
MCU_006785	-2,53	0,0143	21,79	0,0000	Breast cancer type 2 susceptibility protein homolog	DNA binding [GO:0003677]; double-strand break repair via homologous recombination [GO:0000724]	1	0	0
MCU_005442	-2,49	0,0012	22,79	0,0000	Orthologues in cestodes		0	0	0
MCU_007922	-2,46	0,0049	3,31	0,0000	Separin	nucleus [GO:0005634]; cysteine-type endopeptidase activity [GO:0004197]; nuclear chromosome segregation [GO:0098813]; nuclear division [GO:0000280]; proteolysis [GO:0006508]	1	0	1
MCU_009515	-2,46	0,0217	19,53	0,0000	DNA mismatch repair protein Msh2	nucleus [GO:0005634]; ATP binding [GO:0005524]; ATP-dependent DNA damage sensor activity [GO:0140664]; mismatched DNA binding [GO:0030983]; mismatch repair [GO:0006298]	1	0	1
MCU_009565	-2,44	0,0000	6,72	0,0000	Histone H1-beta_late embryonic	nucleosome [GO:0000786]; nucleus [GO:0005634]; DNA binding [GO:0003677]; structural constituent of chromatin [GO:0030527]; nucleosome assembly [GO:0006334]	0	1	0
MCU_005027	-2,40	0,0020	3,35	0,0001	IPR001478:PDZ domain		1	1	0
MCU_012015	-2,40	0,0006	12,33	0,0000	*M. corti* Specific		0	0	0
MCU_012956	-2,37	0,0000	7,74	0,0000	Orthologues in cestodes		0	0	0
MCU_008091	-2,36	0,0101	17,04	0,0000	Rac GTPase-activating protein 1	signal transduction [GO:0007165]	1	0	0
MCU_013359	-2,36	0,0398	15,38	0,0000	*M. corti* Specific		0	0	0
MCU_006326	-2,31	0,0004	20,07	0,0000	Zygotic DNA replication licensing factor mcm3	MCM complex [GO:0042555]; nucleus [GO:0005634]; ATP binding [GO:0005524]; ATP hydrolysis activity [GO:0016887]; DNA binding [GO:0003677]; helicase activity [GO:0004386]; DNA duplex unwinding [GO:0032508]; DNA replication initiation [GO:0006270]	1	0	1
MCU_010783	-2,27	0,0011	12,75	0,0000	Abnormal spindle-like microcephaly-associated protein homolog		1	0	0
MCU_003804	-2,27	0,0000	4,82	0,0000	*M. corti* Specific		0	0	0
MCU_004703	-2,27	0,0118	2,75	0,0001	Transcription factor SOX-8	nucleus [GO:0005634]; DNA binding [GO:0003677]	1	0	0
MCU_007486	-2,22	0,0018	12,71	0,0000	Orthologues in cestodes		0	0	0
MCU_011078	-2,17	0,0066	1,72	0,0335	IPR000198:Rho GTPase-activating protein domain; IPR008936:Rho GTPase activation protein	signal transduction [GO:0007165]	1	0	0
MCU_008718	-2,12	0,0233	10,57	0,0000	Orthologues in cestodes		0	0	0
MCU_004462	-2,11	0,0233	2,96	0,0000	Dynein heavy chain 10_axonemal	cilium [GO:0005929]; dynein complex [GO:0030286]; dynein intermediate chain binding [GO:0045505]; dynein light intermediate chain binding [GO:0051959]; minus-end-directed microtubule motor activity [GO:0008569]; microtubule-based movement [GO:0007018]	0	0	0
MCU_008486	-2,08	0,0032	5,81	0,0000	Telomere-associated protein RIF1		0	0	0
MCU_010900	-2,07	0,0202	2,26	0,0037	*M. corti* Specific		0	0	0
MCU_006812	-2,01	0,0046	27,37	0,0000	DNA replication licensing factor mcm5-A		1	0	1
MCU_007831	-2,00	0,0224	27,84	0,0000	DNA repair protein RAD51 homolog A	ATP binding [GO:0005524]; ATP-dependent DNA damage sensor activity [GO:0140664]; DNA binding [GO:0003677]; DNA repair [GO:0006281]	1	0	1
MCU_004024	-1,98	0,0008	4,16	0,0000	Na- and Cl-dependent neutral and basic amino acid transporter B(0+)	membrane [GO:0016020]; symporter activity [GO:0015293]	0	0	0
MCU_010994	-1,97	0,0089	2,03	0,0389	*M. corti* Specific		0	0	0
MCU_006793	-1,93	0,0210	10,52	0,0000	Tonsoku-like protein		0	0	0
MCU_006062	-1,92	0,0087	16,85	0,0000	DNA replication licensing factor mcm4-B	MCM complex [GO:0042555]; nucleus [GO:0005634]; ATP binding [GO:0005524]; DNA binding [GO:0003677]; DNA helicase activity [GO:0003678]; hydrolase activity [GO:0016787]; DNA replication initiation [GO:0006270]	1	0	1
MCU_003654	-1,92	0,0194	2,89	0,0034	Tyrosine-protein kinase transmembrane receptor Ror2	membrane [GO:0016020]; ATP binding [GO:0005524]; protein tyrosine kinase activity [GO:0004713]; protein phosphorylation [GO:0006468]	0	0	0
MCU_007061	-1,90	0,0036	2,42	0,0013	Palmitoyl-protein thioesterase 1	palmitoyl-(protein) hydrolase activity [GO:0008474]	0	1	0
MCU_002881	-1,89	0,0210	14,15	0,0000	Probable serine/threonine-protein kinase cdc7	ATP binding [GO:0005524]; protein kinase activity [GO:0004672]; protein phosphorylation [GO:0006468]	0	0	0
MCU_009305	-1,85	0,0028	8,74	0,0000	Tubulin alpha chain	microtubule [GO:0005874]; GTP binding [GO:0005525]; hydrolase activity [GO:0016787]; structural constituent of cytoskeleton [GO:0005200]; microtubule-based process [GO:0007017]	0	0	0
MCU_001682	-1,83	0,0101	3,33	0,0000	Rho GTPase-activating protein 19	signal transduction [GO:0007165]	1	1	0
MCOS_0000373901	-1,82	0,0423	16,05	0,0000	IPR022786:Geminin/Multicilin	nucleus [GO:0005634]; regulation of DNA replication [GO:0006275]	0	0	0
MCU_000483	-1,80	0,0282	24,84	0,0000	Histone H2A.J	nucleosome [GO:0000786]; nucleus [GO:0005634]; DNA binding [GO:0003677]; protein heterodimerization activity [GO:0046982]; structural constituent of chromatin [GO:0030527]	1	0	0
MCU_004025	-1,72	0,0125	9,39	0,0000	Importin subunit alpha-1	cytoplasm [GO:0005737]; nuclear import signal receptor activity [GO:0061608]; protein import into nucleus [GO:0006606]	1	0	1
MCU_012071	-3,25	0,0077	-1,68	0,0756	Innexin unc-9	gap junction [GO:0005921]; plasma membrane [GO:0005886]; monoatomic ion transport [GO:0006811]	0	0	0
Downregulated in irradiated worms & with a tendency of enrichment in proliferative cells									
MCU_001783	-2,87	0,0006	1,56	0,0857	IPR008967:p53-like transcription factor, DNA-binding; IPR012346:p53/RUNT-type transcription factor, DNA-binding domain superfamily	nucleus [GO:0005634]; DNA-binding transcription factor activity [GO:0003700]; metal ion binding [GO:0046872]; transcription cis-regulatory region binding [GO:0000976]; apoptotic process [GO:0006915]	1	0	0
MCU_005683	-2,46	0,0210	2,31	0,0925	Mitotic checkpoint serine/threonine-protein kinase BUB1	kinetochore [GO:0000776]; ATP binding [GO:0005524]; protein kinase activity [GO:0004672]; mitotic spindle assembly checkpoint signaling [GO:0007094]; protein phosphorylation [GO:0006468]	0	0	0
MCU_008396	-2,13	0,0023	1,50	0,0847	Cellular tumor antigen p53	nucleus [GO:0005634]; DNA-binding transcription factor activity [GO:0003700]; metal ion binding [GO:0046872]; transcription cis-regulatory region binding [GO:0000976]; apoptotic process [GO:0006915]	1	1	1
Downregulated in irradiated worms & with a tendency of enrichment in differentiated cells									
MCU_000354	-2,41	0,0001	-1,59	0,1039	Nuclear factor interleukin-3-regulated protein		1	1	0
Downregulated in irradiated worms & not differentially expressed in purified cells									
MCU_005413	-135,28	0,0224	5,15	0,3812	Fez family zinc finger protein 2		0	0	0
MCU_003048	-2,90	0,0247	1,09	0,8962	Orthologues in parasites	membrane [GO:0016020]	0	1	0
MCU_008160	-2,67	0,0000	1,01	0,9877	IPR007588:Zinc finger, FLYWCH-type	metal ion binding [GO:0046872]	0	1	0
MCU_008029	-2,05	0,0011	-1,22	0,4924	IPR017877:Myb-like domain	DNA binding [GO:0003677]; DNA-binding transcription factor activity [GO:0003700]	1	1	0
Upregulated in irradiated worms & differentially expressed in purified cells									
MCOS_0000635101	1,83	0,0101	-2,48	0,0006	*M. corti* Specific		0	0	0
MCU_014824	1,98	0,0336	-2,50	0,0058	*M. corti* Specific		0	0	0
MCU_001683	8,39	0,0000	4,59	0,0405	Orthologues in cestodes		0	0	0
Upregulated in irradiated worms & not differentially expressed in purified cells									
MCU_010957	1,67	0,0460	1,34	0,1866	IPR027417:P-loop containing nucleoside triphosphate hydrolase		0	0	0
MCU_009707	1,93	0,0146	1,07	0,8781	*M. corti* Specific		0	0	0
MCU_001950	1,97	0,0006	1,15	0,6639	Orthologues in cestodes		0	0	0

a FC I/C, Fold change: Irradiated vs Control.

b APV, Adjusted p-value.

c FC P/D, Fold change: Proliferative vs Differentiated.

^1^Ortholog associated with stem cell expression in S. mansoni.

^2^Ortholog downregulated by irradiation in H. diminuta.

^3^Ortholog associated with stem cell expression in S. mediterranea.

Among the six upregulated genes in irradiated worms, two unknown *M. corti* specific genes and a conserved unknown gene with orthologues in cestodes are enriched in differentiated cells.

Seven genes differentially expressed in irradiated worms but unchanged in purified cells may be related to the irradiation response of *M. corti*. Three of them are *M. corti* specific unknown genes that may be involved in the different sensitivity to radiation observed in cestodes compared to other flatworms.

These results confirm that irradiation has an effect on proliferative cells, as most of the downregulated genes coincide with genes upregulated in purified proliferative cells.

### 3.7 Several of the upregulated genes in *M. corti* proliferative cells correspond to conserved proliferative markers of model flatworms

**Figure 6:**
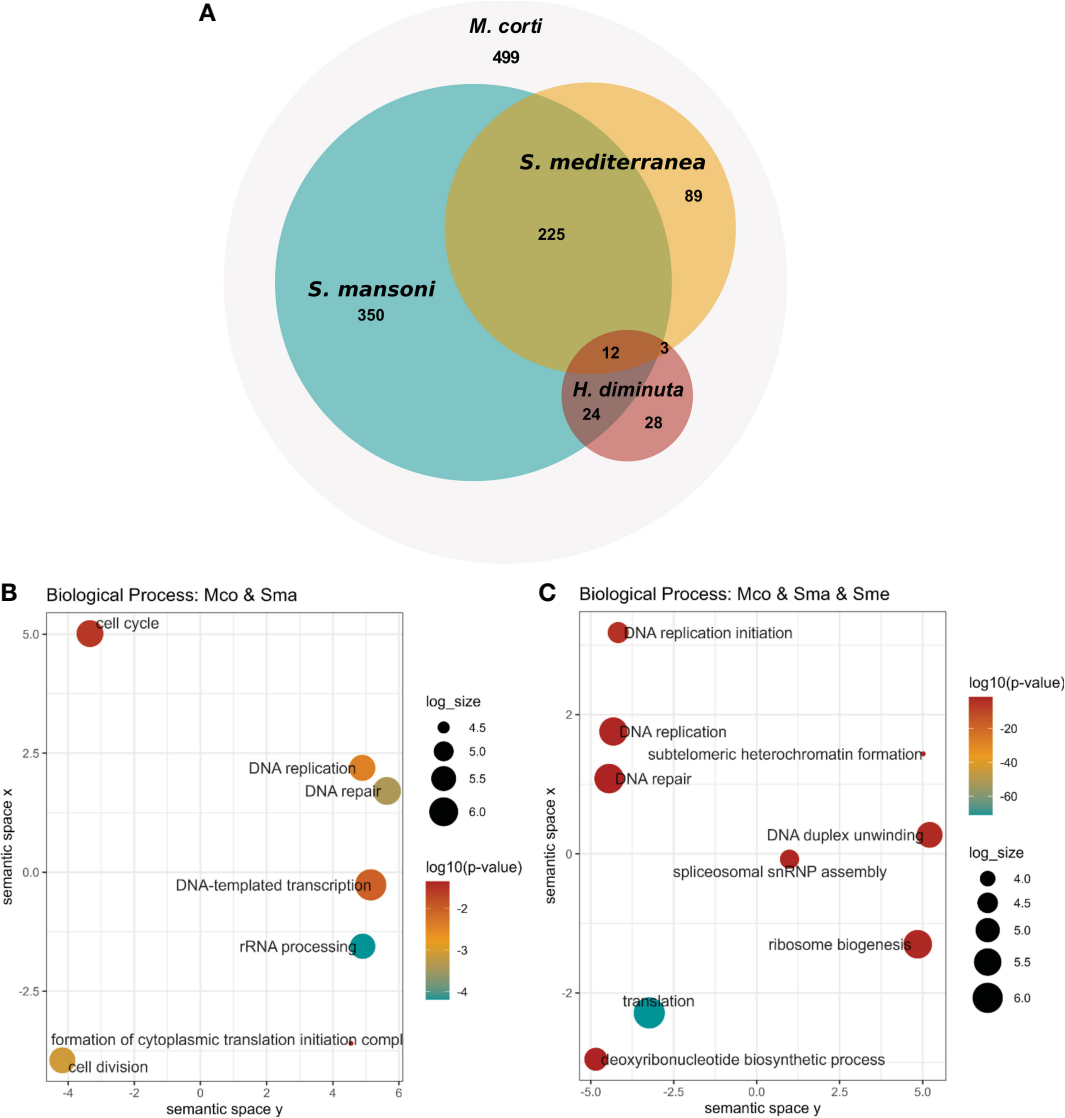
Conservation of stem cell markers in flatworms. **(A)** Number of *M. corti* genes associated with stem cells in at least one work for each organism (see **Supplementary Table 10** for details). **(B)** GO enrichment of conserved genes between *S. mansoni* and *M. corti*. **(C)** GO Enrichment of genes conserved in *S. mansoni*, *S. mediterranea* and *M. corti*.

Upregulated genes in *M. corti* proliferative cells were compared to stem cell markers of other flatworms, as previously done for those downregulated in irradiated worms. *M. corti* proliferative cells shared 611 conserved genes with *S. mansoni*, 329 with *S. mediterranea* and 67 with *H. diminuta*. Twelve genes are shared between the four organisms, and 237 genes are shared between *M.corti*, *S. mansoni* and *S. mediterranea*, species whose stem cells are more studied transcriptomically (**Figure 6A** and **Supplementary Table 10**). The core 237 genes shared between *M. corti* with *S. mansoni* and *S. mediterranea* are enriched in replication machinery elements, cell cycle, RNA biogenesis and translation. This highlights other proliferative functions complementary but distinct from those recovered in irradiated worms, more centered in radiation response and repair (compare **Figures 3**, **6**). Interestingly within the 89 genes shared only between *M. corti* and planaria, splicing, RNA maturation and mitochondria are two terms shared by many genes (**Supplementary Table 10**). On the other hand, those shared only with the trematode *S. mansoni* highlight several genes associated with phosphorylation.

As in irradiated cells, the lower number of shared genes is found between *H. diminuta* and *M. corti* (both cestodes), a feature that might be related to the paucity of cestode data (just two studies including this one). There are 36 genes that are expressed in stem cells of all parasitic flatworms (**Table 2**). No GO terms were found enriched, possibly due to the low number of genes. Beside these, 499 genes upregulated in *M. corti* proliferative cells were not highlighted as differentially expressed in neoblast-like cells of the other flatworms analyzed.

**Table 2:** *M. corti* putative parasitic flatworms stem cell markers.

Gene	Protein Name	Irradiated *M. corti*	Rozario et al., 2019	Wendt et al., 2020	Diaz Soria et al., 2020	Li et al., 2021	Collins et al., 2013	Wang et al., 2013	Collins et al., 2016	Wendt et al., 2018	Fincher et al., 2018	Plass et al., 2018
MCU_005572	PP1-binding domain-containing protein		X	X	X						X	
MCU_007071	BHLH domain-containing protein		X							X		
MCU_003711	Protein kinase domain-containing protein		X				X	X	X	X		
MCU_003748	RMT2 domain-containing protein		X					X		X		
MCU_004472	U2A’/phosphoprotein 32 family A C-terminal domain-containing protein		X	X				X		X		
MCU_001820	60 kDa heat shock protein, mitochondrial		X	X				X		X	X	X
MCU_001017	HMG box domain-containing protein		X	X	X					X		
MCU_000655	Ribonuclease (EC 3.1.26.4)		X					X			X	
MCU_004938	Nuclear pore protein		X					X		X		
MCU_011641	Histone deacetylase complex subunit SAP18		X							X		
MCU_007061	Uncharacterized protein	X	X	X		X						
MCU_008065	Uncharacterized protein		X							X		
MCU_006124	ATP-dependent RNA helicase (EC 3.6.4.13)		X					X		X		
MCU_005265	Dolichyl-diphosphooligosaccharide–protein glycosyltransferase subunit DAD1 (Oligosaccharyl transferase subunit DAD1)		X	X								
MCU_006373	Nop domain-containing protein		X							X		
MCU_006765	Nup54 domain-containing protein		X							X		
MCU_005399	ATP synthase subunit alpha		X		X			X			X	X
MCU_013731	Helicase C-terminal domain-containing protein		X					X				
MCU_008536	BPTI/Kunitz inhibitor domain-containing protein		X			X						
MCU_007715	DUF3504 domain-containing protein		X							X	X	
MCU_001861	Fascin domain-containing protein		X					X		X		X
MCU_010498	C2H2-type domain-containing protein		X				X					
MCU_004085	ATP synthase subunit beta (EC 7.1.2.2)		X					X			X	
MCU_007899	C2H2-type domain-containing protein		X							X	X	
MCU_005569	Kin17_mid domain-containing protein		X							X		
MCU_000059	Ribosomal RNA-processing protein 12-like conserved domain-containing protein		X				X				X	
MCU_006400	SEC7 domain-containing protein		X			X						
MCU_001426	CAF1C_H4-bd domain-containing protein		X							X	X	
MCU_008304	Inactive zinc metalloprotease alpha		X							X		
MCU_005681	SAP domain-containing protein		X					X		X	X	X
MCU_005505	Leucine-rich repeat and WD repeat-containing protein 1 (Origin recognition complex-associated protein)		X							X	X	
MCU_005935	E2F_TDP domain-containing protein		X							X		
MCU_011741	RRM domain-containing protein		X		X			X				
MCU_010047	Dynein light chain		X	X								
MCU_000689	Annexin		X									
MCU_003481	Mitochondrial 28S ribosomal protein S29		X					X				

## 4 Discussion

The exposure of *M. corti* worms to different doses of γ-radiation results in a reduction in proliferative cells, as determined by the incorporation of the thymidine analog EdU. At doses up to 100 Gy, there is a rapid reduction of labeled cells after irradiation, but a recovery to levels similar to unirradiated worms after 5 days, accompanied by no macroscopic damages. Higher doses (500 Gy) result also in early proliferative reduction, but without recovery, and with increasing tegument damage and swelling over time. These results suggest that 100 Gy is a sub-lethal dose for *M. corti*, consistent with observations that proliferative cells in parasitic species might be more resistant to irradiation than planarian neoblasts. While in planarians, a dose greater than 30 Gy is lethal, because all neoblasts are depleted and tissue regeneration is impaired (Wagner et al., 2012; Sahu et al., 2021), doses up to 200 Gy were used as sublethal to study somatic stem cells and identifying irradiation downregulated genes in *S. mansoni* (Collins et al., 2013, 2016; Wendt et al., 2018). Similarly, in the cestode *E. multilocularis*, irradiation with doses up to 150 Gy reduce but not ablate germinative cells, with up to 22% survival, resulting in delayed growth and less proliferation, as well as ultrastructural alterations in the laminated layer (Pohle et al., 2011; Koziol et al., 2014). A similar alteration and a dose dependent increase in apoptosis is observed in *E. granulosus* metacestodes (Alam-Eldin and Badawy, 2015), while in *H. diminuta*, a dose of 200 Gy X-rays reduced proliferative cells after 3 days to 91% and is a lethal dose, as worms were unable to grow and regenerate, leading to worm degeneration after 1 month (Rozario et al., 2019). Even though the required dose seems to be class-dependent, in all flatworms studied, DNA damage induced by radiation results in a decrease of the amount of cells in S-Phase of the cell cycle. Our results are consistent with these observations, indicating that in general parasitic proliferative cells seem to be more resistant to radiation than planarian neoblasts. It should not be ruled out that the structure of the tegument can also favor this resistance (**Supplementary Table 1**).

To verify this, we first analyzed the expression of *McPCNA* and *McNanos*, two genes considered markers of proliferative and germinal cells, respectively. We detected a reduction of their expression after sublethal irradiation, and a recovery close to normal levels after 5 days post treatment. PCNA is involved in DNA replication, and is a good marker gene for proliferating cells, but is also involved in DNA repair (Strzalka and Ziemienowicz, 2011). The increase in mRNA expression observed in worms irradiated with 100 Gy after 5 days of recovery could be explained both by an increase in proliferation or in DNA repair after damage caused by radiation. Nanos is a Zn finger protein that interacts with RNA binding proteins acting as post transcriptional regulator, and has been identified as marker gene for the germ line in planarians and schistosomes (Sato et al., 2006; Handberg-Thorsager and Saló, 2007; Collins et al., 2013; Wang et al., 2013, 2018; Wang et al., 2007; Wendt et al., 2018). These cells should be the most affected because damaged DNA should not be passed to the next generation in gametes. Indeed, we observed that this gene is downregulated after irradiation after one day of recovery.

The analysis of differentially expressed genes in irradiated worms, showed a restricted list of genes, but consistently, several of the downregulated genes were involved with DNA replication or repair, and associated with proliferative cells in other flatworms. In fact, more than half of the *M. corti* downregulated genes are orthologs to genes associated with planarian neoblasts (Fincher et al., 2018; Plass et al., 2018; Zeng et al., 2018), *S. mansoni* neoblast-like cells (Collins et al., 2013, 2016; Wang et al., 2013, 2018; Diaz Soria et al., 2020; Li et al., 2021, 2018; Wendt et al., 2018, 2020) or *H. diminuta* stem cells (Rozario et al., 2019) (**Figure 3C** and **Supplementary Table 6**).

Despite the differences, several genes are shared between flatworms with p53 associated with stem cells in all analyzed species. Parasitic flatworms have two homologs of p53: p53-1 has orthologous genes in several organisms, including human and planaria, while p53-2 is present only in parasitic flatworms (Wendt et al., 2022). In *S. mediterranea*, the unique homolog of p53 is expressed mostly in the neoblast progeny associated with epidermal tissues (Pearson and Sánchez Alvarado, 2010). In *S. mansoni*, p53-1 is expressed in stem cells and tegumental progenitors, while p53-2 has a more diffuse pattern of expression, including stem cells and additional enrichment in the gut and reproductive organs (Collins et al., 2013; Wendt et al., 2020). p53-2 RNAi does not affect tegument or gut but protects proliferative cells from the impact of radiation. It also protects neoblast from chemical genotoxic stress (Wendt et al., 2022). Little is known about the role of these genes in cestodes, so further studies are needed to get a broader picture of their function and the relation with classic p53 function.

Five genes involved in the DNA Damage Response (ATM, BRCA1, BRCA2, BARD1 and RAD51) are downregulated in *M. corti* after irradiation with a sub-lethal dose (100 Gy). BARD1 and BRCA1 are essential for neoblast maintenance in *S. mansoni* (Wendt et al., 2018), while RAD51, ATM and BRCA2 are enriched in planarian neoblasts (Peiris et al., 2016; Sahu et al., 2021). Besides, histone 2AJ downregulated in *M. corti* irradiated worms contains the SQ phosphorylation motif characteristic of Histone 2AX, which is a substrate of ATM (ataxia telangiectasia mutated). In *S. mediterranea* ATM kinase induces apoptosis in cells with DNA damage produced by radiation, as ATM RNAi preserved stem cells after a sublethal dose of radiation. Although ATM preserves stem cells initially after irradiation preventing apoptosis, long term survival depends on homologous recombination DNA repair (mediated by RAD51 and BRCA2) and NHEJ is insufficient to recover cells after irradiation (Shiroor et al., 2023). The planarian homolog of Rad51 is downregulated by lethal doses of radiation and is expressed mostly in neoblasts and is necessary for maintaining DNA and chromosomal integrity. Besides it reduces proliferative cells by half and affects expression of neoblast and progeny marker genes. After irradiation with sublethal doses, Rad51 is downregulated until 3 dpi and increases its expression 5 dpi, which coincides with recovery of mitotic activity (Peiris et al., 2016). Rad51 function is facilitated by BRCA2, and RNAi of this gene produces the same phenotype that Rad51 RNAi (Pearson and Sánchez Alvarado, 2010; Sahu et al., 2021). This implies that RNAi of both components of homologous recombination reduce cell proliferation and alter patterns of cell death.

Since these genes provide a reasonable pathway to explain DNA damage and recovery in the planarian model, we can speculate that they might be basis of the irradiation response in parasitic flatworms However, since these organisms seem to be more resistant to irradiation other genes not shared with planarians could help explain this difference.

Since proliferative cells represent around 7% of the cells of whole worms (Domínguez et al., 2014), it is reasonable that differences at gene expression between irradiated and non-irradiated worms were small. To improve their characterization, we isolated proliferative cells by FACS, and analyzed their transcriptomic profile in comparison with non-proliferative cells. These two cell populations are well differentiated at their transcriptomic level, and segregate consistently with irradiated and non-irradiated worm samples. Differential expression analysis showed more than a thousand genes upregulated in the proliferative (G2/S/M) cell population. Several enrichment analysis procedures showed a more consistent profile in proliferative cells than in differentiated (G0/G1) cells, an expected outcome considering that the G0/G1 cells represent a wide diversity of cell types and tissues. Expectedly, upregulated genes in proliferative cells can be classified in GO terms related to replication, repair, RNA metabolism and cell cycle, and similarly pathways associated with these functions are retrieved by KEGG or Reactome analysis. Most of the genes downregulated in irradiated worms are differentially expressed in proliferative cells, consistent with the well-known idea that irradiation affects proliferation. The comparison of genes identified in proliferative cells of *M. corti* and other flatworms produced large lists of shared genes with *S. mansoni* (Collins et al., 2013, 2016; Wang et al., 2013, 2018; Diaz Soria et al., 2020; Li et al., 2021, 2018; Wendt et al., 2018, 2020) and planarian neoblasts (Fincher et al., 2018; Plass et al., 2018; Zeng et al., 2018), and a more restricted set shared with *H. diminuta* stem cells (Rozario et al., 2019). The quantitative differences might be simply explained by the amount and depth of studies available for non cestode species.

## 5 Conclusion

We used across this study diverse approaches to characterize the proliferative cells of *M. corti*. Despite the differences in sensitivity of these approaches, all of them point consistently to a group of cells responsible for proliferation in this cestode. We showed that although *M. corti* stem cells are sensitive to radiation, the dose needed to ablate them is higher than those needed for other flatworms. Genes downregulated in irradiated worms are mostly associated with DNA metabolism, and particularly with DNA repair, replication and the cell cycle. Consistently most of these genes are upregulated in purified proliferative cells, and shared with those expressed by the stem cells of other flatworms. This is indicative of a common expression profile of proliferative cells across flatworms. In addition to these conserved genes, several *M. corti* specific genes devoid of functional annotation were also differentially expressed. Further functional studies are needed to determine if they are involved in the increased radiation resistance of this species, or if they contribute in a different way to stem cell biology in this model cestode.

## Data availability statement

The data have been deposited with accession numbers PRJNA950029 (irradiation) and PRJNA1039817 (cell purification) in the NCBI BioProject database (https://www.ncbi.nlm.nih.gov/bioproject/).

## Ethics statement

The manuscript presents research on animals that do not require ethical approval for their study.

## Author contributions

AC: Conceptualization, Data curation, Formal analysis, Investigation, Methodology, Validation, Visualization, Writing – original draft, Writing – review & editing. MD: Investigation, Methodology, Writing – review & editing. IG: Investigation, Methodology, Visualization, Writing – review & editing. MP: Investigation, Methodology, Visualization, Writing – review & editing. UK: Investigation, Methodology, Writing – review & editing. EC: Conceptualization, Funding acquisition, Methodology, Project administration, Resources, Supervision, Writing – review & editing. JT: Conceptualization, Funding acquisition, Methodology, Project administration, Resources, Supervision, Validation, Writing – review & editing.

## Supplementary material

Supplementary Figure 1 Worm survival after irradiation.

Supplementary Figure 2 Fold change of marker genes expression in irradiated worms at different recovery times.

Supplementary Figure 3 Enrichment of genes expressed in purified cells.
