# Supplementary material for: Purification and transcriptomic characterization of proliferative cells of Mesocestoides corti selectively affected by irradiation
Source: Front Parasitol. 2024 Mar 5;3:1362199. doi: 10.3389/fpara.2024.1362199 (PMC11732142; doi:10.3389/fpara.2024.1362199)
Supplement: Supplementary file 1 [file DataSheet_1.docx]

# Supplementary Material list and legends

Supplementary material includes 3 supplementary figures and 10 supplementary tables:

*Supplementary Figure 1: Worm survival after irradiation.* Worms exposed to different radiation doses (indicated above the image) recovered at different times (days of recovery are indicated on the left)

*Supplementary Figure 2:* *Fold change of McPUM1, McPUM2 and McpL10 expression in irradiated worms at different recovery times.* Box plots of three replicates per condition. Results after one day post irradiation (1 dpi) are shown in black, while results after 5dpi are shown in gray. p-values from Kruskal-Wallis non-parametric test are shown above each plot.

*Supplementary Figure 3: Enrichment of genes expressed in purified cells.* A-C) GO term enrichment. Circles are sized according to log size and colored by p-value (blue - orange: lower to higher). A) biological process, B) molecular function, C) cellular compartment, D) EggNog functional classification of upregulated genes in differentiated cells (red) or proliferative cells (blue). Bars represent the percentage of differentially expressed genes of the total genes annotated. For further details, (see Supplementary Table 9).

Supplementary Table 1: Effect of different radiation doses in flatworms

Supplementary Table 2: Primer designed for qPCR analysis of candidate marker genes

Supplementary Table 3: Genes associated to stem cells from selected references

Supplementary Table 4: Number of reads and mapping statistics per sample

Supplementary Table 5: GOSeq enrichment analysis of downregulated genes in irradiated worms

Supplementary Table 6: Conservation of downregulated genes in other flatworms

Supplementary Table 7: Number of cells purified, reads and mapping statistics per sample

Supplementary Table 8: (A) Upregulated genes in purified cells (B) Downregulated genes in purified cells

Supplementary Table 9: Enrichment analysis of upregulated genes in purified cells (multipaneled document).

Supplementary Table 10: Full Annotation and conserved neoblast genes (multipaneled document).
