# Supplementary figures and images for: Purification and transcriptomic characterization of proliferative cells of Mesocestoides corti selectively affected by irradiation
Source: Front Parasitol. 2024 Mar 5;3:1362199. doi: 10.3389/fpara.2024.1362199 (PMC11732142; doi:10.3389/fpara.2024.1362199)

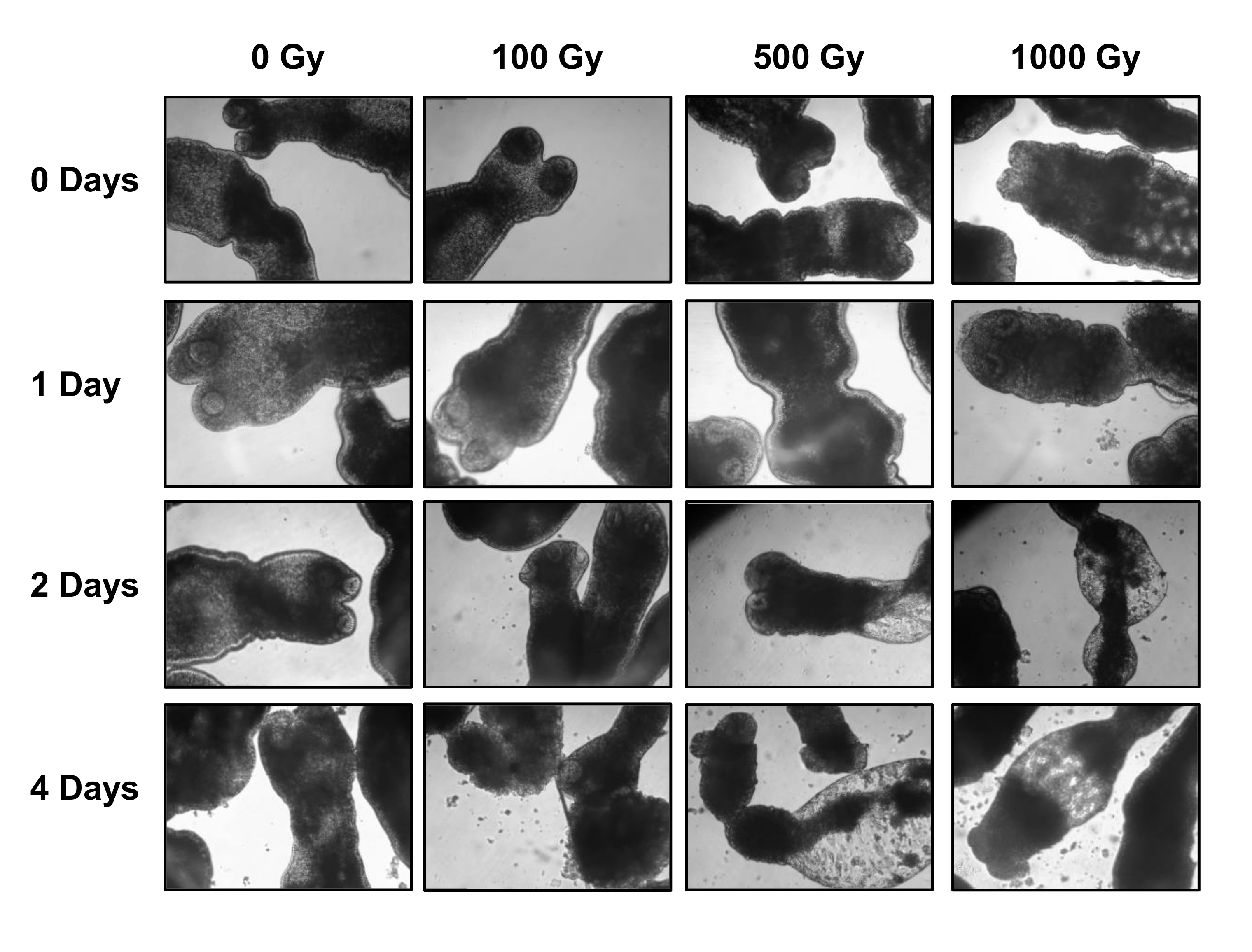

Supplement: Supplementary Figure 1 — Worm survival after irradiation. [file Image_1.tif]

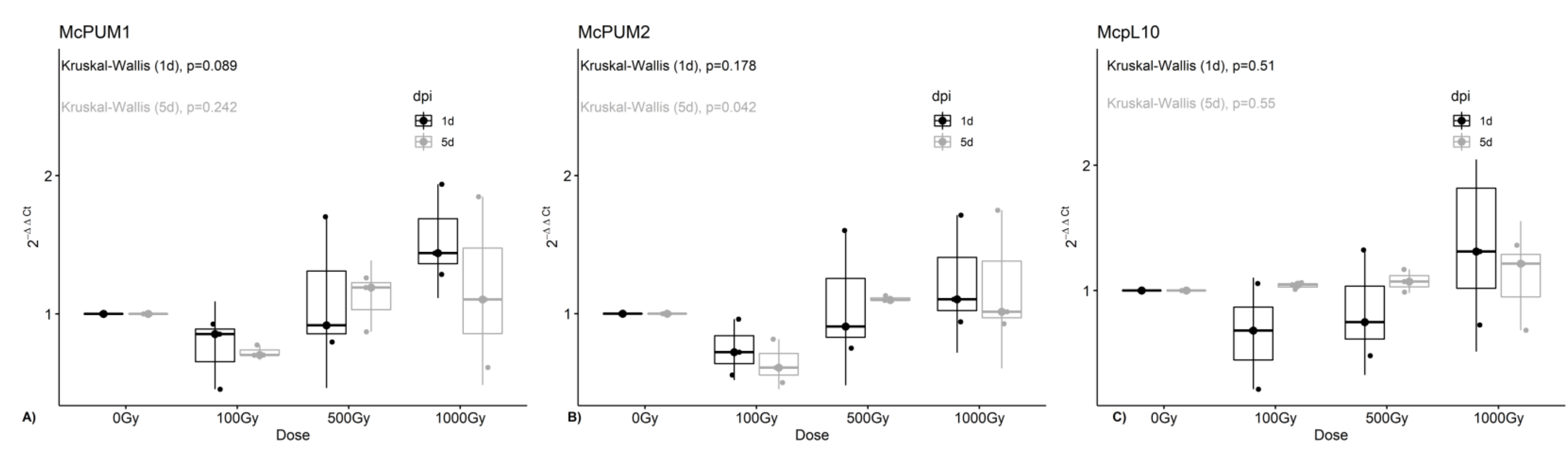

Supplement: Supplementary Figure 2 — Fold change of marker genes expression in irradiated worms at different recovery times. [file Image_2.tif]

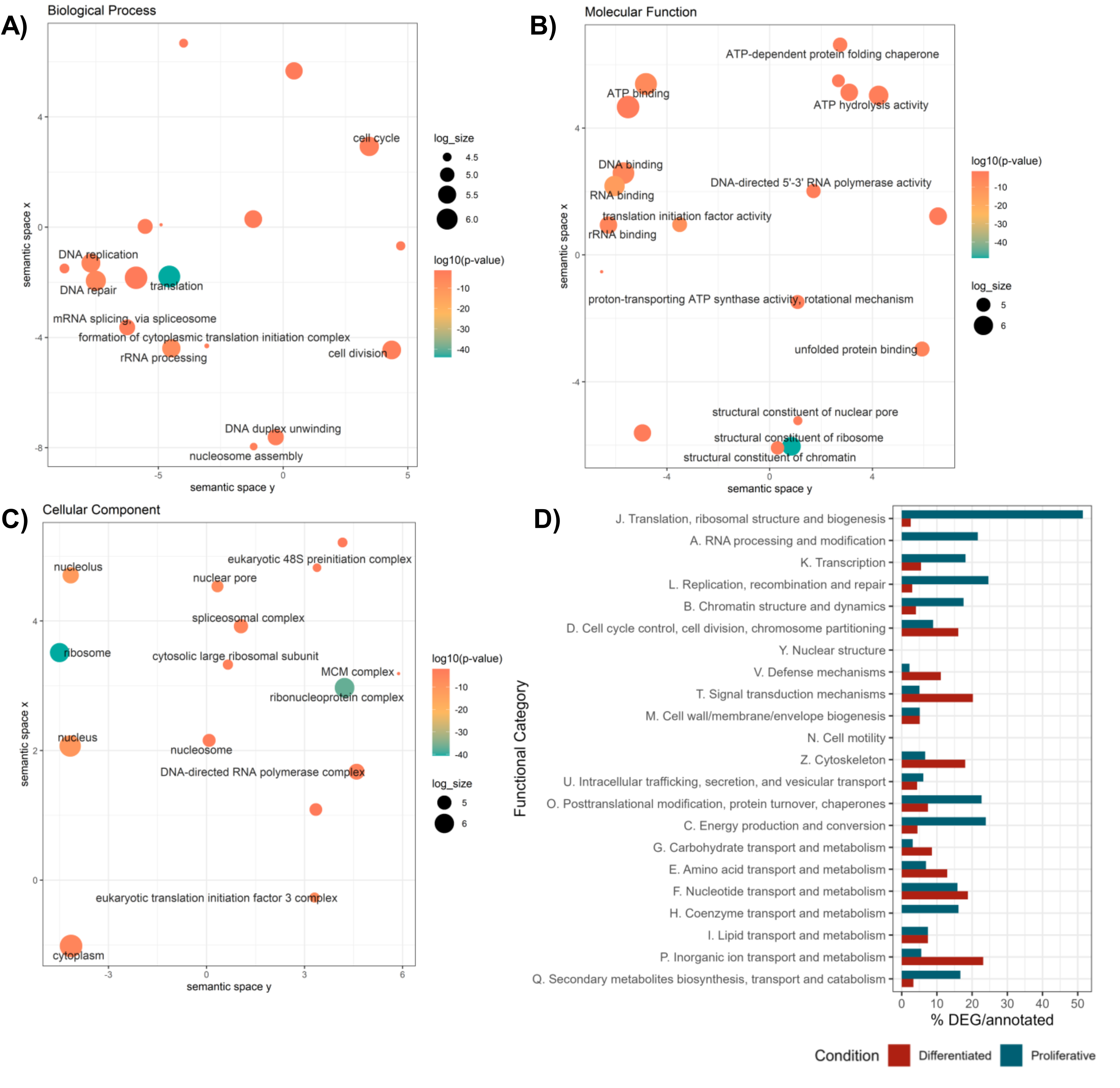

Supplement: Supplementary Figure 3 — Enrichment of genes expressed in purified cells. [file Image_3.tif]
